# Teaching Health Care Disparities in Continuing Medical Education: A Case Report

**DOI:** 10.34067/KID.0000000000000170

**Published:** 2023-05-31

**Authors:** J. Kevin Tucker

**Affiliations:** Division of Renal Medicine, Brigham and Women's Hospital, Mass General Brigham, Harvard Medical School, Boston, Massachusetts

**Keywords:** clinical nephrology, healthcare disparities, medical education

One of the joys of my career as a clinician and educator has been teaching in continuing medical education (CME) conferences. Presenting to an audience in a hotel ballroom or conference center and then meeting participants from around the country and world who ask insightful questions about difficult cases and seeing old friends, who happen to be in the audience as part of the community of learners, added to my personal satisfaction. I will share an experience from a recent teaching experience in a continuing education conference that raised some important teachable moments for me as a clinician-educator.

## The Case

I was invited to give a presentation on CKD in a virtual live-streamed CME conference targeted to primary care providers. In the presentation, I presented several cases and with each one highlighted areas of disparities such as access to kidney transplantation, utilization of home dialysis therapies, and prescription of advanced therapeutic agents such as sodium-glucose cotransporter (SGLT-2) inhibitors. I was surprised to encounter some very harsh, even disrespectful, comments from one among many learners who were engaged in an online question and answer session related to my talk. By reading through the comments, it became clear that the learner was angry that my presentation was not a real talk on CKD because I had weaved throughout the talk lessons on how social determinants of health, race, and yes, racism, affected health outcomes in kidney disease. Fortunately, other learners acted as upstanders who thanked me for the educational content and chided the one learner for being impolite.

## The Virtual Teaching and Learning Experience

The coronavirus disease 2019 (COVID-19) pandemic revealed by necessity that we as educators may deliver high-quality educational content in a distance learning format. The distance learning format also makes the education more accessible to learners who otherwise might not be able to participate because of the costs of travel or the inability to be physically away from the workplace or family for several days. Not every eager learner has a CME budget provided by his or her employer.

However, after experiencing online vitriol, I also reflected that in an in-person event, it is likely that no learner would have approached me with those comments. As in the world of social media, the relative anonymity of virtual questions, answers, and comments may remove the traditional guardrails against incivility. Other learners—as happened in the case cited—must be upstanders who help to enforce standards of discourse in the online learning environment.

## Why Teach about Health Care Disparities?

At about the same time that we were understanding that distance learning can be quite effective, the murder of George Floyd and other persons of color at the hands of law enforcement, coupled with the stark racial and ethnic differences in morbidity and mortality from COVID-19, underscored the need to understand more deeply the root causes of health care disparities. These root causes include not only the usual social determinants of health that we may have learned in medical school or residency but also structural racism. Undergraduate and graduate medical educators as well as those of us who regularly teach our professional colleagues re-examined curricula to ensure that there was appropriate discussion of health care disparities, not as a separate and unequal otherwise obligatory disparities talk but embedded within the curriculum for each clinical entity being discussed.^1^

As a nephrologist who has seen firsthand the impact of health care disparities, I have made a deliberate effort over the past 2 years to include content that underscores the relationship between social determinants of health, race, and racism on important health outcomes. Furthermore, in the milieu of an academic bubble, I had never been made to feel that these topics were controversial or unimportant.

For practitioners in the community who are on the frontlines in providing patient care, it is imperative that they understand the barriers to equal care. Consider as an example that SGLT-2 inhibitors are now known to slow the progression of kidney disease in patients living not only with diabetes but also with other forms of CKD. It makes no difference that SGLT-2 therapies may slow the progression of CKD if poor and marginalized patients are not being offered those therapies because of unconscious biases or because they cannot afford them. But that is precisely what is happening. Black and Latinx patients are prescribed SGLT-2 inhibitors at significantly lower rates than White patients.^2^ That is not to accuse clinicians of deliberately choosing not to prescribe these newer medications at the same rate. There are myriad barriers including insurance and copays among others, but we also must worry that implicit bias may be creeping in.

This possibility of bias is not unique to the prescription of SGLT-2 inhibitors. Racial differences in prescriptions of direct oral anticoagulants have also been noted,^3^ and in an academic medical center, Black and Latinx patients have been found to be less likely to be admitted to a specialized cardiology service, relative to White patients with the same presenting complaints.^4^

I questioned the content that I had chosen to present and began to doubt myself as a teacher. Did I emphasize disparities too much at the expense of bread-and-butter nephrology content?

The course directors did their best to restore my confidence as a teacher and reassured me that the vitriolic comments were largely coming from one individual. Nonetheless, I still wondered if I should eliminate the lessons on disparities in kidney disease. With the passage of a few weeks, I am now firm in my resolve to continue to include content related to health care disparities and their underlying drivers in my teaching.

## Action Steps for Nephrology Educators

The steps that we as nephrology educators must take to ensure that our education is holistic in its approach may be guided by the principles of our specialty societies. The American Society of Nephrology's Statement Against Racism states that “Racism prevents ASN from fulfilling its mission to prevent, treat, and cure kidney diseases and to advance the highest quality of care for patients. ASN joins the Council of Medical Specialty Societies and other associations in opposing racism, addressing health disparities and social determinants of health, and promoting diversity, equity and inclusion.”

- Learning objectives: Learning objectives should be examined through the lens of addressing health disparities and promoting diversity, equity, and inclusion by, for example, including at least one learning objective that aims to close the knowledge gap with respect to health care disparities in kidney disease.
- Curricula: Nephrology curricula should include case studies with real-world examples that illustrate how bias, social determinants of health, race, and racism affect the care of patients living with kidney disease.
- Faculty diversity: Our faculty need to represent the diversity within the specialty. At the same time, racial and ethnic minorities remain woefully underrepresented in academic medicine. Therefore, we must commit to training fellows from underrepresented backgrounds, encouraging them to pursue academic careers and striving to retain them as faculty.
- Upstander training: Knowing how to respond and support learners who experience racism or microaggression is an essential tool for creating a supportive learning environment. Toolkits are readily available to learn these important skills to support our learners as they deal with episodes of bias.^5^
- Antiracism: Nephrology educators must work with their divisions, departments, hospitals, and health systems to be proactively antiracist by dismantling the structures of systemic racism that underlie health disparities.^6^ An example in kidney medicine relates to creation of vascular access for hemodialysis. For members of marginalized communities, the lack of transportation may be a significant structural barrier that impedes creation of a vascular access. One academic medical center has created a Saturday vascular access clinic which makes vascular access creation less onerous for working people or those who rely on working family members for transportation.^7^ More of these creative strategies are needed to address the structural barriers to equitable care.

For seasoned clinicians of my vintage, for whom teaching about health care disparities in medical school and residency was very limited and superficial, the continuing education environment is critical for raising awareness and understanding if we are to ever achieve more equitable health outcomes. Understanding systemic barriers, including our own implicit biases, to equitable care in CKD is just as important—if not more important—for the primary care provider as understanding anemia of CKD.

Societal progress is often not linear. We move forward, but then there is a backlash, and we move backward. As clinicians suffer unprecedented degrees of burnout in the aftermath of COVID-19 and a seemingly unrelenting mismatch between the demand for health care and capacity, it is not surprising that those on the frontlines may become irritable with education that seems to them at first blush superfluous and an impingement on their personal time. Some state medical boards, for example, the Massachusetts Board of Registration in Medicine, have taken the bold stance of requiring implicit bias training for licensure. Let us always remind ourselves and those whom we educate, however, that the goal is simply to create more just and equitable outcomes.

## Disclosures

J.K. Tucker reports the following: Ownership Interest: Altria, Kraft-Heinz, Mondelez International, Norfolk Southern, Philip Morris International, Regions Bank, and Southern Company, and Other Interests or Relationships: UpToDate—Peer Reviewer.
